# d-Lysergic Acid Diethylamide (LSD) as a Model of Psychosis: Mechanism of Action and Pharmacology

**DOI:** 10.3390/ijms17111953

**Published:** 2016-11-23

**Authors:** Danilo De Gregorio, Stefano Comai, Luca Posa, Gabriella Gobbi

**Affiliations:** 1 Neurobiological Psychiatry Unit, McGill University, Montreal, QC H3A 1A1, Canada; danilo.degregorio@mail.mcgill.ca (D.D.G.); luca.posa@mail.mcgill.ca (L.P.); 2 Division of Neuroscience, San Raffaele Scientific Institute and Vita-Salute University, 20132 Milan, Italy; comai.stefano@hsr.it

**Keywords:** LSD, psychosis, serotonin, dopamine, TAAR_1_, hallucinogens, atypical antipsychotics

## Abstract

d-Lysergic Acid Diethylamide (LSD) is known for its hallucinogenic properties and psychotic-like symptoms, especially at high doses. It is indeed used as a pharmacological model of psychosis in preclinical research. The goal of this review was to understand the mechanism of action of psychotic-like effects of LSD. We searched Pubmed, Web of Science, Scopus, Google Scholar and articles’ reference lists for preclinical studies regarding the mechanism of action involved in the psychotic-like effects induced by LSD. LSD’s mechanism of action is pleiotropic, primarily mediated by the serotonergic system in the Dorsal Raphe, binding the 5-HT_2A_ receptor as a partial agonist and 5-HT_1A_ as an agonist. LSD also modulates the Ventral Tegmental Area, at higher doses, by stimulating dopamine D_2_, Trace Amine Associate receptor 1 (TAAR_1_) and 5-HT_2A_. More studies clarifying the mechanism of action of the psychotic-like symptoms or psychosis induced by LSD in humans are needed. LSD’s effects are mediated by a pleiotropic mechanism involving serotonergic, dopaminergic, and glutamatergic neurotransmission. Thus, the LSD-induced psychosis is a useful model to test the therapeutic efficacy of potential novel antipsychotic drugs, particularly drugs with dual serotonergic and dopaminergic (DA) mechanism or acting on TAAR_1_ receptors.

## 1. Introduction

### 1.1. LSD: “A Joyous Song of Being” (Albert Hoffman)

d-Lysergic Acid Diethylamide (LSD) was first synthesized in 1937 by Albert Hoffman [1]. LSD produces changes in body perception, synaesthesia, thought disorders, and time distortion [2]. LSD has potent psychotropic effects, described as inducing “mystical experiences” [3]; alterations of the state of consciousness, euphoria, enhanced capacity for introspection, altered psychological functioning, a sense of unity, transcendence of time and space, and positive mood; feelings of joy, blessedness and peace; a sense of sacredness; and a positive attitude towards others and the self [2,4,5,6]. It stimulates the sympathetic system, causing hyperthermia, sweating, palpitation, the elevation of blood pressure, convulsions, an increase in muscle tension, tremors, and muscular incoordination [7,8,9]. Hoffman described his first experience with LSD as like “a travel in the universe of the soul, a waves of ineffable happiness flowed through his body”. In his memoir, he wrote: “I had experienced the grace of God” and the feeling that of “God is in everything” [1]. One of the main effect of LSD is indeed the mystical experience and the “transcendence of time and space”, meaning that the subject feels himself to be beyond past, present, and future, and beyond ordinary three-dimensional space; the subject feels himself to exist in a realm of eternity or infinity [10].

Besides inducing this sort of mystical experience and psychotic-like effects in healthy subjects, LSD—particularly among vulnerable people and at high dose—may also rarely induce lasting psychosis. This particular characteristic invited basic scientists and clinicians to explore the pharmacology of LSD and the transient LSD-induced psychotic-like state. LSD’s remarkable potency led psychiatrists to speculate about the existence of an endogenous LSD-like “schizotoxin” in the brain of patients with schizophrenia [11].

This review attempts to summarize research on the mechanism of action of LSD-induced psychosis.

### 1.2. Psychosis and LSD: Human Studies

Generally speaking, LSD at relatively high doses produces a state of transient psychotic-like state, but in some vulnerable subjects can produce a psychosis. In this section, we summarize the studies reporting hallucinogenic and psychotic-like effects of LSD as well the occurrence of psychosis induced by LSD.

Psychosis is characterized by a certain degree of impairment in social functioning and the experience of several psychopathological symptoms such as hallucinations, avolition or apathy, catatonic motor behaviour, disorganized behaviour, disordered thoughts manifested by speech, flat or inappropriate affect, depersonalization/derealisation, or delusions [12,13,14]. Depending on their content, delusions are classified in *persecutory*, *grandiose*, *erotomanic*, *nihilistic*, or *somatic* (American Psychiatric Association’s *Diagnostic and Statistical Manual of Mental Disorders*, *Fifth Edition* (*DSM-5*)) [15]. Religious delusions and/or pathological hypereligiosity is another symptom of psychosis; interestingly, this symptom has likewise been observed in schizophrenia associated with temporal epilepsy [16]. When psychotic symptoms last for at least six months and include at least one month of active-phase symptoms, the diagnosis of schizophrenia can be made. In schizophrenia, hallucinations, delusions, illusions, and disorganized thinking are classified as positive symptoms, while blunted affect, emotional withdrawal, and apathetic social withdrawal are classified as negative symptoms [17]. LSD represents the first synthetic drug whose effect, particularly at high doses, mimics some symptoms of psychosis and schizophrenia, inducing a transient psychotic-like state; for this reason, much research has been performed on the mechanism of action of this drug, as its pharmacology could potentially shed light on the pathophysiology of psychosis and schizophrenia. LSD can also induce a distortion of space or time that is typical of psychotic experiences. [18]. Other symptoms and experiences that link LSD to a psychotic-like state (at a medium dose of 100–200 µg *per os* (p.o.)) include: a metamorphosis-like change in objects and faces, a metamorphic alteration of body contours, a change in body image, and intense (kaleidoscopic or scenic) visual imagery with transforming content, deficit in sensorimotor gating [5]. Studies aimed at comparing hallucinogen-induced psychotic states with the early stages of psychosis have confirmed a substantial degree of overlap between LSD-induced psychosis and early symptoms of schizophrenia, like hallucinations, conceptual disorganization, and unusual thoughts [19,20].

A list of LSD’s most relevant effects related to psychotic-like symptoms and a comparison with symptoms of psychosis are reported in Table 1.

However, in vulnerable subjects (people with a pre-existent risk of mental disease), LSD may induce a real psychosis, described as panic, paranoia and distrust, suspicious feelings or delusions of grandeur, confusion, impairment of reasoning, regret, depression, loneliness and/or somatic discomfort, all of which can be of monumental proportions [21].

Klee and Weintraub [22] reported that individuals presenting some psychological characteristics including mistrustfulness, complaining, fearfulness, and susceptibility on projection as a defence were more likely to show paranoid symptoms during the LSD experience in comparison with healthy people. One study in healthy volunteers [23] observed that pre-drug indicators on the Rorschach test of paranoid features and the potential for a thought disorder predicted the advent of psychotic-like experiences after the consumption of LSD. Of particular note, LSD-induced psychotic symptoms were more pronounced in 18 out of 20 relatives of schizophrenics [24], suggesting that persons with a greater genetic predisposition to schizophrenia are more susceptible to an LSD-induced psychotic response, as likewise suggested by studies of cannabis [25,26]. In keeping with these results, Vardy and Kay [27] suggested that LSD-induced psychosis was a drug-induced schizophreniform reaction, and that there was a greater response to LSD in individuals with a genetic predisposition to schizophrenia.

Similarly, Ungerleider et al. [28] found that symptoms of schizophrenia, measured with the Minnesota Multiphasic Personality Inventory (MMPI), are more common among LSD users with pre-existent prolonged psychosis, and concluded that “LSD interacts with schizoid traits”. Using the MMPI, Smart and Jones [29] examined 100 LSD users and 46 non-users and found a much higher incidence of psychopathology among LSD users than non-users. The MMPI scale revealed a profile of alienation, emotional disturbances, excessive anxiety, depression, hallucinations, paranoia, and suicidal tendencies in LSD users. A study by Tucker and colleagues [30] which compared hospitalization due to LSD and other hallucinogenic-drug casualties with that of schizophrenic inpatients found that the LSD/hallucinogenic group had higher associative productivity, intrusion of primitive drive content, and penetration scores than schizophrenics. Taken together, these studies suggest that LSD may interact with premorbid schizophrenic vulnerability to yield psychosis [31].

Cohen et al. [32] found that several psychiatric reactions, including psychotic breaks and panic attacks, occurred in 0.8 per 1000 healthy volunteers, and in 1.8 per 1000 psychiatric patients. These reactions lasted over 48 h. While no successful suicides or suicide attempts were reported in healthy volunteers [32], they occurred in less than 0.4 per 1000 patients. This lack of LSD-induced psychosis and suicidality in the general population was recently confirmed by an extensive epidemiological study [33].

There have been observed correlations between recreational LSD use and psychological, perceptual, and cognitive dysfunction: impaired performance on tests of visual perception and spatial orientation, the occurrence of magical thinking, decreased ability to perform well on tests of non-verbal abstract reasoning, and hallucinogen persisting perception disorder (HPPD) [33,34,35,36,37]. There are also correlations between LSD use and prolonged psychotic decompensation, paranoid reactions, depression, the exacerbation of psychopathy and antisocial personality traits, and neuroleptic malignant syndrome [38]. A recent study by Carhart-Harris et al. [39] examined the acute and mid-term psychological effects of LSD, and found that LSD produced strong psychological effects such as elevated mood and high scores on the Psychotomimetic States Inventory (PSI), an index of psychosis-like symptoms. 

Only a few studies have investigated the treatment of LSD-induced psychosis. In patients admitted to the emergency room with a diagnosis of acute phencyclidine- or LSD-induced psychosis, Giannini et al. [40] showed that haloperidol (5 mg, intra-muscular (i.m.)) and chlorpromazine (50 mg, i.m.) were effective in reducing psychotic-like symptoms, but haloperidol produced a greater degree of improvement compared to chlorpromazine on ratings of anxiety, cognitive and conceptual disorganization, mannerisms, posturing, and visual hallucinations. On the other hand, chlorpromazine was found to be more effective than haloperidol at reducing somatic and psychological tension. While behavioural animal studies have examined the effect of chronic treatment with LSD [41], no research has been conducted yet to examine long-term behavioural changes in healthy individuals after repeated administration of LSD. This lack of long-term studies is likely due to the evidence that LSD is a drug taken occasionally by recreational users that does not produce addiction.

**Figure 1:**
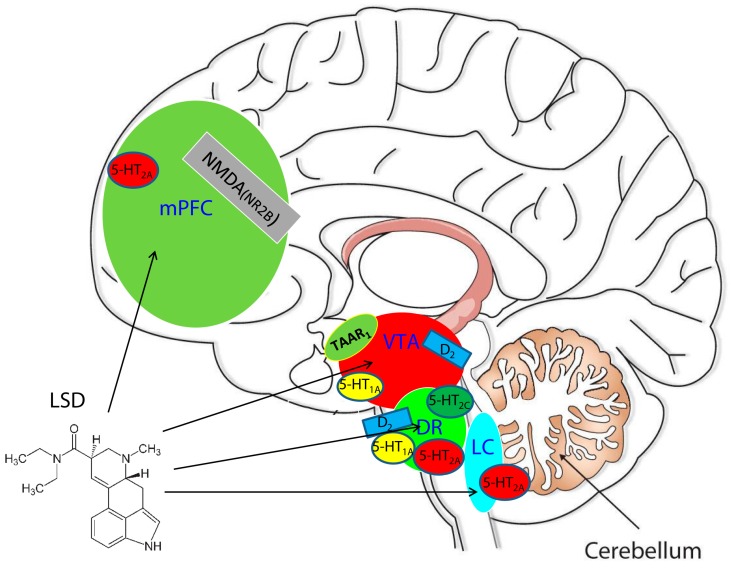
d-Lysergic Acid Diethylamide (LSD) acts at different brain regions with a pleiotropic mechanism of action involving serotonin 5-HT_1A_, 5-HT_2A_, 5-HT_2C_, and dopamine D_2_ receptors in the Dorsal Raphe (DR); dopamine D_2_ receptor and Trace Amine Associate (TAAR_1_) receptors in the Ventral Tegmental area (VTA); and 5-HT_2A_ in the Locus Coerules (LC). These three nuclei project to the prefrontal cortex (PFC), enhancing or inhibiting the release of neurotransmitters and ultimately medicating the psychotic-like effects and cognitive changes. mPFC: medial prefrontal cortex (mPFC); NMDA(NR2B): *N*-methyl-d-aspartate (NMDA) receptor subunit NR2B.

Intriguingly, recent neuroimaging studies carried out by the group of Nutt and colleagues [42] reveal marked changes in brain activity after LSD administration that strongly correlate with LSD’s characteristic psychological effects. Employing different brain imaging techniques including resting state MRI and magnetoencephalography, the researchers measured blood flow, functional connections within and between brain networks, and brainwaves in the volunteers on and off the drug [42]. LSD’s effects on the brain’s visual cortex did not correlate significantly with its other established effects on consciousness. On the other hand, LSD administration decreased signalling between the parahippocampus and the retrosplenial cortex (RSC); the magnitude of the decrease correlated with ratings of “ego-dissolution” and “altered meaning”, both of which are considered hallmarks of psychosis. In particular the ego dissolution may correspond to the dissociative identity disorder described by Schneider in schizophrenic patients [43]. Of note, the researchers observed significant associations between findings in different imaging techniques, enabling firmer inferences about the functional significance of these brain networks, including how the networks’ functioning can model certain pathological states [42]. This recent, unique, and comprehensive examination of consciousness following LSD administration represents an important advancement in the field of psychedelics, particularly in a time of growing interest in their scientific and therapeutic value. The LSD-induced psychotic state has been historically attributed to its agonist activity at serotonin receptors; its pharmacology led researchers to suspect that LSD could provide a preclinical model of psychosis [41,44]. This concept prompted the fundamental hypothesis that abnormalities in serotonergic function are responsible for psychosis spectrum disorders, including schizophrenia and schizophreniform disorder. The inference was that modulators of serotonin receptors might be useful in the treatment of schizophrenia [45,46]. Multiple lines of evidence indicate that LSD acts through the serotonergic system, binding the 5-HT_2A_ receptor as a partial agonist, even at human cloned 5-HT_2A_ receptor [47,48,49,50,51], and the 5-HT_1A_ receptor as an agonist/partial agonist [52,53]. However, studies have also pointed out the involvement of the dopaminergic [50,54] and glutamatergic [55] systems in LSD’s mechanism of action. Burt and colleagues demonstrated in 1975 that LSD has binding affinity for labelled ^3^H-dopamine and ^3^H-haloperidol binding sites [54]. More recent work demonstrated an interaction between LSD and the D_2_ receptor [56,57,58]. Intriguingly, an in vitro study by Bunzow et al. in 2001 [59] demonstrated the affinity of LSD for the trace amice-associate receptor 1 (TAAR_1_), a G-protein coupled receptor discovered in 2001 [59]. Preliminary studies hypothesized that TAAR_1_ receptors are implicated in the pathogenesis of psychosis by interacting with the dopaminergic system [60,61,62]. Given the evidence that abnormal dopaminergic function is implicated in psychosis [63,64], the relationship between D_2_, TAAR_1_ and LSD’s psychotic-like effects deserves further consideration in terms of both the neurobiology and the neuropsychopharmacology of psychosis. In this context, here we review current preclinical and clinical knowledge on the involvement of the serotonergic, dopaminergic, glutamatergic, and trace-amine neurotransmission in the psychotic-like state induced by LSD. A graphical interaction of LSD with these neurotransmissions is reported in Figure 1.

## 2. The Dopamine-Serotonin System and TAAR_1_ Receptor in the Pathogenesis of Psychosis and in the Mechanism of Action of Antipsychotic Drugs

It is well known that drugs possessing antipsychotic activity have mostly dopaminergic and/or serotonergic activity. The therapeutic mechanisms of conventional typical antipsychotic drugs, such as haloperidol, depend on their blockade of the brain’s D_2_ receptors, in particular at the level of mesolimbic dopamine (DA) pathway, which reduces hyperactivity in this pathway and thus, the positive symptoms of psychosis [65,66,67]. However, D_2_ receptors in the mesolimbic DA system mediate not only psychotic positive symptoms, but also the brain’s reward system, in particular the nucleus accumbens—which is considered the brain’s “pleasure centre” [68]. Unfortunately, D_2_ receptor typical antipsychotics act not only in the mesolimbic DA pathway, but also in the nigrostriatal DA pathway, producing extrapyramidal symptoms and tardive dyskinesia [69]. In addition, the blockade of D_2_ receptors in the tuberoinfundibular DA pathway is the cause of hyperprolactinemia [70].

The newer atypical antipsychotic drugs mostly act instead on 5-HT receptors, which underlines the role of interactions between the serotonergic and dopaminergic systems in the pathogenesis and treatment of psychosis [71]. Binding affinity studies find strong interactions between atypical antipsychotics and the 5-HT_2A_ and 5-HT_1A_ receptors [72]. Some serotonin receptors have a major influence on dopamine release; specifically, when serotonin is released in the vicinity of postsynaptic 5-HT_2A_ receptors, dopaminergic neurons are inhibited, providing a braking action on dopamine release. On the contrary, the 5-HT_1A_ receptor is an accelerator of dopaminergic function, because its inhibition of serotonin release prevents 5-HT_2A_ postsynaptic receptors on dopamine neurons from being activated [72]. The therapeutic efficacy of classical antipsychotic drugs is supposed to require an occupancy of dopamine D_2_-receptors >70% [73]. However, when atypical antipsychotic drugs are used in clinical settings, they seem effective even at subthreshold occupancies of D_2_ receptors [74,75], likely thanks to their interaction with 5-HT_1A_ and 5-HT_2A_ receptors in the medial prefrontal cortex (mPFC) [76,77]. Negative symptoms and cognitive deficits in schizophrenia have been associated with reduced dopaminergic function in the mPFC [71]. Thus, one treatment strategy is the development of therapeutics that promote increased dopaminergic function in the mPFC [71]. Atypical antipsychotic drugs increase dopamine release in the mPFC through a mechanism dependent on postsynaptic 5-HT_1A_ receptors [78,79]. This effect could be useful in the treatment of both negative and cognitive symptoms [80,81,82] compared to typical antipsychotics. Of note, several studies reported a beneficial effect of 5-HT_1A_ agonists in reversing and preventing the development of catalepsy in rodents [83,84], suggesting that the combination of a 5-HT_1A_ agonist and a D_2_ antagonist may lead to antipsychotic activity free of extrapyramidal symptoms [82]. Other animal studies have confirmed a role for 5-HT_2_ receptors in alleviating catalepsy through the use of specific 5-HT_2A_ antagonists [85,86] and have also shown that 5-HT_2_ antagonists enhance dopamine-mediated motor behaviour in models other than catalepsy [87,88]. Though Kapur and Seeman [89] have recently argued in their PET studies that the occupancy of 5-HT_2A_ is not a necessary condition for atypicality, PET occupancy metrics may not be directly correlated to drugs’ antipsychotic mechanism of action. Indeed, as reported by Cox and colleagues, PET studies allow a maximum resolution area of 4.2 mm [90]; this low resolution makes it difficult to study the restricted and small areas where antipsychotics ultimately act, such as the ventral tegmental area (VTA) [91,92].

In recent years, the TAAR_1_ receptor has been identified as a novel therapeutic target in the treatment of psychotic disorders. Discovered in 2001 [59,93], TAAR_1_ is an important modulator of the dopaminergic and serotonergic systems [94,95,96,97,98], and potentially the glutamatergic system [94]. TAAR_1_ is a G protein-coupled receptor that responds to the so-called trace amines (TAs), a subgroup of biogenic amines including β-phenylethylamine (PEA), *p*-tyramine (pTyr), and tryptamine previously denoted as false neurotransmitters [59,93,95,96,97,99]. Abnormal levels of TAs has been associated with various neuropathological disorders, including schizophrenia [33,67,68,69,70,71], major depression [71,72] and Parkinson’s disease [63,95,98,99,100]; in addition, the TAAR_1_ gene maps to locus 6q23 have been frequently associated with schizophrenia and bipolar disorder [100,101]. In the murine brain, TAAR_1_ is expressed in the limbic and monoaminergic systems, including the VTA and dorsal raphe nucleus (DRN) [60]. Mice lacking TAAR_1_ receptors (TAAR_1_^−/−^ mice) display no overt phenotype, but they are hypersensitive to amphetamine, showing enhanced locomotion, and striatal release of DA, noradrenaline (NA), and 5-HT following an acute challenge with the psychoactive drug [60,102]. In addition, the basal spontaneous firing activity of VTA DA and DRN 5-HT neurons in these knockout mice is augmented and does not decrease following injection of pTyr compared with WT controls [60,94,103]. These results suggest that TAAR_1_ can be considered a modulator of monoaminergic neurotransmission; it also suggests potential relevance for the development of novel therapeutics targeting this receptor in the context of neuropsychiatric disorders. The recent development of potent TAAR_1_ antagonists such as EPPTB and RO5212773 [103,104] and the TAAR_1_ agonist RO5166017 [94] will allow exploration of this possibility. In vitro studies with these ligands have revealed that TAAR_1_ functionally interacts with D_2_ and 5-HT_1A_ receptors on VTA DA and DRN 5-HT neurons, respectively [94,103,105]. In vivo, TAAR_1_ agonists produce anxiolytic and antipsychotic-like effects in several mouse models [94]. Importantly, similarly to the antipsychotic olanzapine, RO5166017 induces a reduction of the DA-dependent hyperlocomotion induced by cocaine administration or genetic deletion of the DA transporter, as well as hyperactivity caused by L-687,414, an antagonist of the *N*-methyl-d-aspartate (NMDA) receptor [94].

## 3. In the Deep of the Mechanism: Who Are the Players?

### 3.1. The Serotonin System and LSD: The Playmaker or the Point Guard

Similar to basketball games, several players are involved in the pharmacology of LSD.

**Figure 2:**
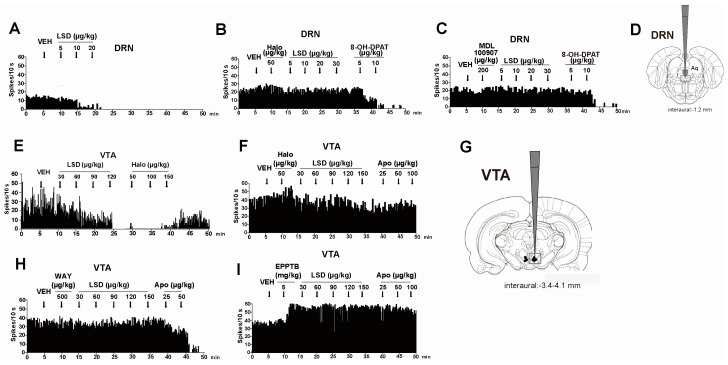
Representative integrated firing rate histograms showing the effects of intravenous LSD administration on the firing rate of dorsal raphe nucleus (DRN) serotonin (5-HT) neurons and of ventral tegmental area (VTA) dopamine (DA) neurons (modified from De Gregorio et al. 2016, with permission [115]): (**A**) LSD (5–20 µg/kg) decreases DRN 5-HT neurons; (**B**) Haloperidol (halo, 50 µg/kg); and (**C**) MDL 100 907 (200 µg/kg) prevent the inhibitory effect of LSD on DRN 5-HT neurons; (**D**) illustration portrays a coronal brain section (Paxinos and Watson, 1986 [117]) containing the DRN, the boxed area encompassing the DR represents the location where most putative 5-HT neurons were found; (**E**) LSD (30–120 µg/kg) decreased VTA DA neurons: (**F**) Haloperidol (halo, 50 µg/kg) prevents the inhibitory effect of LSD on VTA DA neurons (**G**) illustration portrays a coronal brain section (Paxinos and Watson, 1986) containing the VTA; (**H**) WAY 100 907 (WAY, 200 µg/kg); and (**I**) EPPTB (5 mg/kg) prevent the inhibitory effect of LSD on VTA DA neurons.

In both humans and rodents, there is a strong correlation between the binding affinities of LSD-like hallucinogenic drugs at the 5HT_2A_ receptor and their hallucinogenic potencies [50,106,107,108]. Notably, the activation of the 5-HT_2A_ receptor may promote the experience of visual hallucinations, likely by increasing neuronal excitability and altering visual-evoked cortical responses [109]. The effects of LSD on the 5-HT inhibition observed in electrophysiological studies have been correlated with their human hallucinogenic effects [110]. It has also been shown that the selective 5HT_2_ antagonists LY 53857 and ritanserin can reverse the inhibitory effect of systemic administration of LSD on the spontaneous activity of Locus Coeruleus (LC) neurons [110]. Following this evidence, the 5HT_2A_ receptor has been designated as being responsible for LSD-induced hallucinations [106,107,111]. Marek et al. [112] have demonstrated that low concentrations of LSD (3–100 nM) excite GABAergic interneurons in the layer III of the rat pyriform cortex, and this effect is blocked by the selective 5-HT_2A_ antagonist MDL-100,907. Many other studies have likewise highlighted the involvement of other serotonin 5-HT_1A_ and 5HT_2C_ receptor subtypes in the mechanism of action of LSD [52,53,113]. The 5-HT_2A_ and 5-HT_2C_ receptors have similar molecular and pharmacological properties, and the phenylisopropylamine hallucinogens as well as LSD have comparable affinities for these serotonin receptor subtypes [114]. Studies conducted to understand the selective role of serotonin receptors in the mechanism of action of hallucinogenic drugs suggest that these drugs primarily act via 5-HT_2A_ receptors, with 5-HT_1A_ and 5-HT_2C_ receptors having modulatory roles [112,113,114]. In particular, acute administration of the selective serotonin re-uptake inhibitor (SSRI) citalopram potentiates the stimulus effects of the phenethylamine (−)-2,5-dimethoxy-4-methylamphetamine (DOM), and this effect was reversed by the selective 5-HT_2C_ receptor antagonist SB-242084. Electrophysiological studies performed in our laboratory demonstrate that the cumulative injection of LSD (30–150 µg/kg, intra-venous (i.v.)) significantly decreases the firing rate of DA VTA neurons in rats and that this effect is blocked by the previous injection of WAY-100 635 (500 µg/kg, i.v.), a selective 5H_1A_ antagonist (Figure 2E,H). We also confirmed LSD’s inhibitory effect (5–20 µg/kg) on 5-HT neurons in the Dorsal Raphe nucleus (DRN), which was prevented by the injection of the selective 5-HT_2A_ antagonist MDL 100 907 (200 µg/kg) (Figure 2A,C) [115]. Fiorella et al. [116] found that depleting 5-HT in rats using *p*-chlorophenylalanine (PCPA) resulted in the supersensitivity of LSD-entrained animals to the stimulus effects of LSD, which was likely caused by a significant upregulation (+46%) of the maximal level of 5-HT_2C_ receptor-mediated phosphoinositide hydrolysis. Similarly, we found that 5-HT depletion with PCPA (350 mg/kg, intra-peritoneal (i.p.) per day, for two days) does not affect the inhibitory effect of LSD on the firing activity of VTA DA neurons per se, but that it sensitizes the LSD-induced burst response, shifting the curve to the left [115].

Krall et al. [118] investigated the LSD-induced stimulus control in Serotonin Transporter Knockout (SERT KO) mice. The efficacy of the stimulus control induced by LSD in these animals is markedly decreased. The authors speculated that the reduced density of 5-HT_1A_ and/or 5-HT_2A_ receptors observed in these knockout mice underlies the absence of stimulus control by LSD. However, the potential interaction between LSD and SERT remains controversial. In fact, in a recent study Kyzar et al. [119] examined heterozygous SERT^+/−^ mouse self-grooming and several other stereotypic behaviours following acute LSD administration (0.32 mg/kg), finding that SERT^+/−^ mice, compared to controls, display a longer duration of self-grooming behaviour, but that treatment with LSD increases serotonin-sensitive behaviours, such as head twitching, tremors, and backwards gait in both SERT*^+/+^* controls and SERT*^+/−^* mice. These data suggest LSD’s SERT-modulating effect on specific behaviours; however, a direct interaction between LSD and SERT should be excluded, as demonstrated by Richli et al. [50] in in vitro affinity studies. It is likely that the decreased stimulus effect observed by Krall et al. [118] was related to a reduced density of 5-HT_1A_ and/or 5-HT_2A_ receptors in these knockout mice (as the authors speculated). Overall, more studies are necessary to further elucidate the link between serotonin receptors and the effects of LSD. A detailed summary of the interaction of LSD with the serotonergic system is reported in Table 2.

### 3.2. The Dopamine System: The Power Forward

Burt and colleagues conducted a pilot study to test the possible interaction of LSD with the dopaminergic system in 1975. They found that LSD displays highly stereospecific binding to the dopamine (DA) receptor, with the d-isomer displaying about 1000 times greater affinity than the l-isomer for both labelled 3H-dopamine and 3H-haloperidol binding sites [54]. Later, the hypothesis that the dopaminergic system could be involved in the effects of LSD caught the attention of other researchers. LSD binds to the D_1_ and D_2_ receptors as a partial agonist [120], and the D_4_ receptor as a full agonist [124]. In particular, LSD shows high affinity (*k*_i_ = 2 nM) for D_2_ receptors in both pig brain [57] and in human cloned D_2_ receptors [50,59]. Giacomelli et al. investigated the functional activity of LSD at DA receptors in primary cultures of rat pituitary cells in their study of prolactin secretion. In this model, LSD produced a dose-dependent inhibition of prolactin secretion in vitro with an IC_50_ of 1.7 nM. This effect is antagonized by spiperone, but not by SKF83566 or cyproheptadine, indicating that LSD’s inhibition of prolactin secretion was mediated by D_2_. Interestingly, LSD, at 10^−13^–10^−10^ M, potentiates prolactin secretion induced by dopamine in pituitary cells in vitro [127]. On the other hand, human studies performed by Schmid et al. have demonstrated that LSD induces an increase in prolactin secretion via the 5-HT system [9]. These findings suggest that LSD not only interacts with DA receptors, but also modulates DA neurotransmission. Seeman et al. also demonstrated that LSD and phencyclidine induce the incorporation of [^35^S]GTPγS into the inhibitory G protein (G_i_) coupled to D_2_ receptors in homogenates of rat brain striatum [56]. These in vitro studies were confirmed in vivo in male rats: LSD (0.05 and 0.20 mg/kg) significantly decreases plasma prolactin levels. In addition, 0.20 mg/kg of LSD inhibited the increase of plasma prolactin levels produced by chlorpromazine (5 mg/kg) and alpha-methylparatyrosine (50 mg/kg). LSD’s effects were more potent than methysergide, a serotonin receptor blocker, in lowering plasma prolactin levels, and more potent than apomorphine in blocking the increase in plasma prolactin produced by quipazine, a 5-HT agonist [128]. Rats that had received the D_1_/D_2_ agonist apomorphine (0.25 mg/kg) respond to LSD with partial generalization [121]. However, the apomorphine cue was antagonized by the D_2_ antagonist haloperidol, and not by the 5-HT antagonist pizotifen; further, the substitution of LSD for apomorphine was blocked by pizotifen, but not haloperidol. These findings suggest that serotonin rather than dopamine is implicated in the substitution process. Notably, the mixed 5-HT_2A_/D_2_ antagonist risperidone blocked the LSD cue with much greater potency (414 times) than the 5-HT_2_ antagonist ritanserin [122,129]. At a dose of 0.63 mg/kg ritanserin produces full occupation of 5-HT_2A_ receptors, but fails to attenuate the LSD stimulus. On the contrary, at a much higher dose (40 mg/kg), which produced significant occupation of catecholamine receptors, ritanserin completely antagonized the LSD cue [122,130]. Unlike the LSD cue, the DOM cue is antagonized by risperidone and ritanserin with nearly identical potencies [128]. These experiments suggest that 5-HT_2A_ and D_2_ receptors are implicated in the LSD discrimination, whilst only the 5-HT_2A_ receptor is involved in DOM discrimination. As further support, the potency of ritanserin at antagonizing LSD stimulus control is enhanced when co-administered with low doses of the D_2_ antagonist haloperidol [122]. For these reasons, the LSD cue seems most effectively and potently antagonized by concurrent blockade of 5-HT_2A_ and DA receptors. The authors found that risperidone antagonizes the LSD cue with a potency comparable to that deriving from the co-administration of ritanserin and haloperidol [122]. Interestingly, recent reports indicate that the LSD discriminative stimulus being 5-HT- or DA-mediated is actually just a matter of time [123]. Drug discrimination studies using LSD as the training drug and 15–30 min pre-treatment times consistently demonstrated that 5-HT_2A_ antagonists block LSD-induced stimulus control. On the other hand, when using a longer pre-treatment time (90 min), the resulting stimulus cue evoked by LSD is mediated by D_2_-like but not 5-HT_2A_ receptors [124,131]. Therefore, the stimulus effects of LSD follow two distinct temporal phases involving 5-HT_2A_ receptors in the first phase and D_2_ receptors in the second phase [132]. Even though a previous clinical study highlighted the important role of the 5-HT_2A_ receptor to reduce the psychotomimetics effects of psilocybin, independently of D_2_ stimulation [133], future studies should clarify whether the delayed dopaminergic cue is a direct effect of LSD or relies on a metabolite of LSD with selective D_2_ agonist activity. In accordance with these findings, we found that cumulative injections of LSD (5–120 μg/kg, i.v.) significantly decreased the firing rate of 5-HT DRN and DA VTA neurons of rats and that this effect was prevented by the injection of the selective D_2_ antagonist haloperidol (50 µg/kg) (Figure 2A,B,E,F) [115]. Intriguingly, Martin et al. recently found that the cortical DA system appears to be persistently affected by chronic treatment with LSD (0.16 mg/kg per day, 90 days) in rats [134]. In particular, they observed a 40% decrease in the mRNA expression of the DA receptor genes *Drd1* and *Drd2* in the medial Prefrontal Cortex (mPFC), suggestive of a receptor downregulation. As the authors speculated, that might be due to repeated excess dopaminergic activity in the frontal cortex following chronic LSD administration. LSD may be acting directly on the receptors, or it may act through indirect modulation of DA release, mediated by 5-HT_2A_ receptor activation in the mPFC [135]. Our recent electrophysiology studies suggest a direct activation on D2, TAAR1 receptors in the dopaminergic neurons of the VTA [115]. 

A detailed summary of the interaction of LSD with the dopaminergic system is reported in Table 2.

### 3.4. Glutamate: The Third Player or Small Forward

The first experiment regarding the role of glutamate in the molecular functioning of LSD was carried out by Aghajanian and Marek in 1999 [55]. They demonstrated that LSD and phenethlylamine hallucinogens (e.g., 1-(2,5-dimethoxy-4-iodophenyl-2-aminopropane) (DOI)), though they partially act through 5-HT_2A_ receptors, enhance a prolonged and late wave of glutamate release onto layer V pyramidal neurons in the rat prefrontal cortex after an excitatory postsynaptic current (EPSCs) [54]. More recently, Lambe and Aghajanian [125] found that the NMDA receptor subunit NR2B-selective antagonists, ifenprodil and Ro25-6981, suppress this LSD- and DOI hallucinogen-induced delay in glutamate release and that this effect could also be partially mimicked by inhibiting glutamate uptake. This finding suggests that hallucinogenic drugs induce an over-release of glutamate in a phasic manner, unlike the glutamate uptake inhibitors [125]. Since a hyper-glutamatergic state may be involved in prodromal stages of schizophrenia [136], NR2B antagonists could be useful in the treatment of this pathological state. Moreno et al. have explored the effect of the chronic treatment with the mGlu2/3 receptor antagonist LY341495 (1.5 mg/kg) on the hallucinogenic-like effects induced by LSD (0.24 mg/kg). They found that the head-twitch behavior and the expression of c-fos, egr-1, and egr-2 were decreased by the administration of LY341495, revealing that the blockade of the mGlu2 receptor reduced the hallucinogenic effects of LSD due to 5-HT_2A_ receptor activation [126].

A detailed summary of the interaction of LSD with the glutamatergic system is reported in Table 2.

### 3.5. Trace Amine-Associated Receptor 1 (TAAR_1_) and LSD: The Shooting Guard

Psychostimulant and hallucinogenic amphetamines, numerous ergoline derivatives including ergometrine, dihydroergotamine, and LSD, as well as the antiparkinsonian agents bromocriptine and lisuride, display agonistic activity at the rat [59] and mouse [51] TAAR_1_ receptor expressed on HEK-293 cells. Simmler et al. showed that LSD has relatively high affinity for TAAR_1_ in rat but lower in mice and humans [137], for the first time, our laboratory showed that the injection of EPPTB (5 mg/kg, i.v.), a novel and selective TAAR_1_ antagonist, prevents the inhibitory effect of LSD (30–150 µg/kg, i.v.) on rat VTA DA firing activity (Figure 2I) [115]. These studies suggest that TAAR_1_ receptor may play at least a downstream role in the response to LSD, which deserves further study since TAAR_1_ may be a novel target for the treatment of psychosis and LSD-induced psychotic-like effects.

A detailed summary of the interaction of LSD with the TAAR_1_ receptor is reported in Table 2.

### 3.6. Prefrontal Cortex: The Center

The final pathways of the serotonergic, dopaminergic, TAAR_1_ and glutamatergic activation by LSD is the Prefrontal Cortex (PFC). Projections from DR nuclei, VTA and LC end in the PFC promoting or blocking the release of neurotransmitters [138]. Indeed, the PFC contains pyramidal glutamatergic neurons that are modulated by several systems as well as γ-aminobutyric acidergic (GABA) interneurons and dopaminergic, noradrenergic, serotonergic, glutamatergic and cholinergic neurotransmitters. Any change in these systems in the PFC could lead to the development of altered behavioural phenotypes [139]. Most of the psychotic and cognitive effects of LSD are probably mediated by the PFC [140]. However, further research should be addressed to explore the involvement of PFC in the mechanism of LSD.

## 4. LSD: An Animal Model of Psychosis

Several behavioural experiments have been carried out in animals to better understand LSD effects in laboratory settings. Chronic administration of LSD (0.08 and 0.16 mg/kg per day for three months) in rats induces a variety of persistent abnormal behaviours including hyperactivity, hyper-reactivity, abolished preference for sucrose solution, and altered social behaviours [42] that persist for several months after discontinuation. In particular, increased locomotion is present for at least three months after LSD cessation, and both olanzapine and haloperidol temporarily attenuate this phenomenon [41]. Drug-discrimination (DD) paradigms are used to examine the abuse-related effects of drugs by establishing the interoceptive effects of a training drug as the cue for performing a specific operant response (e.g., the pressing of a lever). Details on the DD protocol can be found in Solinas et al. [141]. Marona-Lewicka et al., using a two-lever food-reinforced operant conditioning task in rats, found that the discriminative stimulus effect of LSD occurred in two temporal phases, with the first phase mediated by 5-HT_2A_ receptors, and the second one mediated by D_2_-like DA receptors [123,132]. Hallucinogenic 5-HT_2A_ agonists including LSD and mescaline produce deficits in startle habituation (a psychotic-like symptom) in rats [142], while 5-HT_2A_ antagonists [143], including antipsychotics like clozapine and clothiapine [144], yield opposite behavioural effects. Chronic treatment with LSD induces widespread changes in the neuronal state at the level of the mPFC that is present even four weeks after treatment cessation [134]. Importantly, LSD leads to altered expression of proteins and genes involved in schizophrenia. Indeed, LSD increases mRNA expression for the NMDA receptor subunit NR2A, the growth factor Bdnf, Krox20, and GABA-A ion channels (Gabrb1), and decreases RNA expression for the DA receptor gene *Drd2*, the two cytochrome C oxidase IV subunit genes (*Cox7a2*, *Cox8a*), and for glutathione *S*-transferases (Gstt2, Gstp2) [134]. Prepulse inhibition of the startle response (PPI) is considered a model of the gating deficits in schizophrenia and is used in animal models to test psychotic-like symptoms [145]. Indeed, clinical studies report that symptoms related to PPI deficits are common in schizophrenic patients [146,147,148]. Previous studies revealed that LSD produced disruption of PPI in rodents [149,150] and humans [9]. In particular, the PPI-disruptive effects of LSD are blocked by the selective 5-HT_2A_ antagonist M100907, but not by the D_2_ blocker haloperidol [143]. The head-twitch behavioural response is characterized by a rapid and lateral movement of the head that is similar to the pinna reflex [151]. It has been shown to be induced by a variety of psychedelic 5-HT_2A_ receptor agonists such as LSD, DOI, mescaline, and psilocin [152,153,154], and reversed by 5-HT_2A_ receptor antagonists [153,155,156]. Moreno et al. [157] found that the head-twitch response induced by LSD in mice is decreased by chronic treatment with clozapine (25 mg/kg/day, for 21 days), an atypical antipsychotic with low affinity for the D_2_ receptor, suggesting a predominant role of the 5-HT_2A_ receptors over the D_2_ receptors in the mechanism of action of LSD.

## 5. Conclusions

LSD is a very complex molecule, acting through multiple targets, whose chronic administration leads to the development of psychotic-like symptoms. Although there is a paucity of recent human studies and clinical reports concerning LSD-induced psychotic effects, preclinical and clinical studies suggest that LSD-induced psychosis may represent a valid model of psychosis that may allow researchers to investigate the pathogenesis of psychosis and the effectiveness of novel antipsychotic drugs that act through synergistic effects on the serotonergic and dopaminergic systems. LSD-induced psychosis in laboratory animals may also allow the investigation of the preclinical efficacy of novel antipsychotic drugs including TAAR1 ligands. Clinical research with LSD, psilocybin, and other phenethylamine derivatives is currently undergoing a major revival, and current studies are exploring the potential role of hallucinogens in the treatment of alcohol dependence [158] as well as mood disorders [159] and anxiety [160]. In particular, a recent study carried out by Dolder et al. has demonstrated that the administration of 100 µg of LSD enhances emotional empathy in healthy volunteers [161] However, more research needs to be performed on the differential effects of LSD and other hallucinogenic drugs at low and high doses: while at lower doses they activate 5-HT system, likely producing therapeutic effects for depression, drug dependence [159], and anxiety [160], it is clear that at high doses they activate the dopaminergic system, producing psychosis and similar effects [115].

## Figures and Tables

**Table 1:** List of the most relevant effects induced by LSD related to a psychotic-like syndrome (left) and symptoms of psychosis (right). (Modified after: Passie et al., 2008 [5] and Schmid et al., 2015 [9]).

Parallelism between Effects Induced by LSD (100–200 µg) and Symptoms of Psychosis	
LSD-Induced Symptoms	Psychosis or Schizophrenia
Metamorphic alterations, unusual inner perception of bodily processes and changes in body image	Body Distortion
Metamorphosis-like change in objects and faces and intense (kaleidoscopic or scenic) visual imagery with transforming content	Delusions
Changes in perception and sensory alteration: Visual, auditory, taste, olfactory, kinaesthetic (pseudo-hallucinations)	Hallucination (Visual, auditory, taste, olfactory, kinaesthetic)
Depersonalization, derealisation	Depersonalization, derealisation, Cotard’s syndrome
Alteration of affectivity: euphoria, mood swing, anxiety	Euphoria, Dysphoria, Depression, Blunted affect
Mystical experience	Religious delusion, hypereligiosity
Suicide attempts	Depression and Suicide
Introjection	Interoception
Broader and unusual association	Clang association
Hyporeactivity	Psychomotor retardation, Catatonia
Disruption of sensorimotor gating	Deficit in sensorimotor gating
Disruption of pre-pulse inhibition (PPI)	Impairment in prepulse inhibition (PPI) of the acoustic startle response
Attention span shortened, alteration of Thinking, memory changes and decreased non-verbal abstract reasoning	Severe cognitive and memory impairments, Working memory impairment
Flash-back phenomena	“Déjà-vu” experiences

**Table 2:** Summary of the experiments showing the interaction of LSD with serotonergic, dopaminergic, glutamatergic and TAAR systems.

Results of LSD’s Effect on Serotonin, Dopamine, Glutamate and TAAR Systems		
	In Vivo Studies	In Vitro Studies
5-HT_1A_	5-HT_1A_ receptor agonists increase the effect of LSD (0.1 mg/kg) in stimulus control test in rats. The effect is reverted by the 5-HT_1A_ antagonist WAY 100 635 [53]	Radio-labelled [3H]-LSD binds 5-HT_1A_ receptors (*k_i_*: 1.1 nM) in homogenates of rat cerebral cortex [52]
	5-HT_1A_ receptor antagonist WAY 100 635 (500 µg/kg) prevents the inhibitory effect of LSD (30–150 µg/kg) on VTA DA firing activity [115]	LSD binds human cloned 5-HT_1A_ (*k_i_*: 0.0030 ± 0.0005 µM) receptor in HEK 293 cells [50]
5-HT_2A_	LSD (3–100 nM) excites GABAergic interneurons in the layer III of rat pyriform cortex; the effect is blocked by the 5-HT_2A_ antagonist MDL 100 907 [112];	Binding assay reveals affinity of radio labelled [3H]-LSD for 5-H_2A_ receptor (*k_i_*: 2.5 nM) in parietofrontal cortex of male rats [5]
	5HT_2_ antagonist LY 53857 reverts the inhibitory effect of systemic administration of LSD (5–10 µg/kg) on the spontaneous activity of Locus Coeruleus (LC) neurons in rats [110]	
	5-HT_2A_ antagonist MDL 100 907 (200 µg/kg) prevents the inhibitory effect of LSD (5–20 µg/kg) on DRN 5-HT firing activity [115]	LSD binds Human 5-HT2A receptor-expressing NIH-3T3 cells (*k_i_*: 0.0042 ± 0.0013 µM) [50]
5-HT_2c_	serotonin depletion with PCPA induces supersensitivity of LSD(0.1 mg/kg)-trained rats to the stimulus effects of LSD and upregulation of the maximal level of 5-HT_2C_ receptor [116]	Labelled LSD binds 5-HT_2C_ receptor (*k_i_*: 10 nM) in NIH-3T3 cells transfected with rat 5-HT_2C_ receptor [5];
		LSD binds human cloned 5-HT2c (*k_i_*: 0.015 ± 0.003 µM) receptor in HEK 293 cells [50]
Serotonin Transporter (SERT)	The efficacy of the stimulus control induced in SERT KO mice reveals a decreased efficacy of LSD (0.17–0.30 mg/kg)-stimulus control [118];	LSD did not interact with SERT in HEK 293 cells [49]
	SERT^+/−^ mice compared to controls display a longer duration of self-grooming behaviour. The treatment with LSD (0.32 mg/kg) increases serotonin-sensitive behaviours such as head twitching, tremors and backwards gait in both SERT*^+/+^* controls and SERT*^+/−^* mice [119]	
D_1_	No available studies	Labelled LSD binds D_1_ receptor (*k_i_*: 27.1 nM) in C-6-mD_1A_ cells of rat striatum [120];
		LSD binds human cloned D_1_ (*k_i_*: 0.31 ± 0.1 µM) receptor in HEK 293 cells [50]
D_2_	Rats trained with the DA D_1_/D_2_ agonist apomorphine (0.25 mg/kg) respond to LSD with partial generalization but the apomorphine cue is antagonized by the D2 antagonist haloperidol [121]	LSD stimulates the incorporation of [^35^S]GTP-γ-S into G_i_ coupled to D2 receptors in homogenates of rat brain striatum [56]
	The potency of ritanserin to antagonize LSD stimulus control is markedly potentiated when administered in combination with low doses of the D_2_ antagonist haloperidol [122]	LSD displaces the selective D_2_ antagonist [^3^H]raclopride from pig brain cryostat sections with an IC_50_ of 275 Nm [57]
	LSD (372 nmol/kg, 0.16 mg/kg) injected 90 min before training produces a cue that is not fully blocked by 5-HT_2A_ antagonists, but instead is significantly inhibited by the D_2_ antagonist haloperidol [123]	LSD stimulates the incorporation of [^35^S]GTP-γ-S into G_i_ coupled to human cloned D_2_ receptors [58]
	Haloperidol (50 µg/kg) prevents the inhibitory effect of LSD (30–150 µg/kg) on VTA DA firing activity [115]	LSD binds human cloned D_2_ (*k_i_*: 0.025 ± 0.0004 µM) receptor in HEK 293 cells [50,51]
D_4_	The D_4_ antagonist A-381393 attenuates the stimulus effect of LSD (0.08–0.016 mg/kg) in discriminative test [124]	^38^ L Labelled LSD shows affinity for NIH3T3 fibroblast cells expressing the rat D_4_ receptor (*k_i_*: 56 nM) [5]
NMDA	NMDA receptor subunit NR2B-selective antagonists, ifenprodil and Ro25-6981 suppress the prolonged glutamate release induced by LSD onto layer V pyramidal neurons of the prefrontal cortex [125]	No available studies
mGlu_2_/mGlu_3_	mGlu2/3 receptor antagonist LY341495 (1.5 mg/kg) reduces head-twitch behaviour and expression of c-fos, egr-1 and egr-2 that are both augmented by LSD (0.24 mg/kg) [126]	No available studies
TAAR_1_	The selective TAAR_1_ antagonist EPPTB (5 mg/kg) prevents the inhibitory effect of LSD (30–150 µg/kg) on VTA DA firing activity [115]	LSD binds the expressed rat TAAR_1_ receptor on HEK-293 cells (*k_i_*: 0.8 µM) [59];
		LSD binds rat (*k*_i_: 0.45 ± 0.05 µM) and mouse (*k*_i_: 10 ± 2.9 µM) TAAR_1_ receptor in HEK 293 cells [51]

## Abbreviations

p.o.	per os
i.v.	intra-venous
i.m.	intra-muscular
i.p.	intra-peritoneal

## References

[1] Hofmann A., Ott J. (1980). LSD, My Problem Child.

[2] Savage C. (1952). Lysergic acid diethylamide (LSD-25) A Clinical-Psychological Study. Am. J. Psychiatry.

[3] Pahnke W.N., Richards W.A. (1966). Implications of LSD and experimental mysticism. J. Relig. Health.

[4] Hensala J.D., Epstein L.J., Blacker K. (1967). LSD and psychiatric inpatients. Arch. Gen. Psychiatry.

[5] Passie T., Halpern J.H., Stichtenoth D.O., Emrich H.M., Hintzen A. (2008). The pharmacology of lysergic acid diethylamide: A review. CNS Neurosci. Ther..

[6] Matthew H. (1968). Lysergic acid diethylamide intoxication. Br. Med. J..

[7] Goodman N. (2002). The serotonergic system and mysticism: Could LSD and the nondrug-induced mystical experience share common neural mechanisms?. J. Psychoact. Drugs.

[8] Dawson P., Moffatt J.D. (2012). Cardiovascular toxicity of novel psychoactive drugs: Lessons from the past. Prog. Neuropsychopharmacol. Biol. Psychiatry.

[9] Schmid Y., Enzler F., Gasser P., Grouzmann E., Preller K.H., Vollenweider F.X., Brenneisen R., Müller F., Borgwardt S., Liechti M.E. (2015). Acute effects of lysergic acid diethylamide in healthy subjects. Biol. Psychiatry.

[10] Pahnke W.N. (1967). LSD and Religious Experience. LSD Man & Society.

[11] Osmond H., Smythies J. (1952). Schizophrenia: A new approach. Br. J. Psychiatry.

[12] Hartley S., Barrowclough C., Haddock G. (2013). Anxiety and depression in psychosis: A systematic review of associations with positive psychotic symptoms. Acta Psychiatr. Scand..

[13] Faerden A., Barrett E.A., Nesvåg R., Friis S., Finset A., Marder S.R., Ventura J., Andreassen O.A., Agartz I., Melle I. (2013). Apathy, poor verbal memory and male gender predict lower psychosocial functioning one year after the first treatment of psychosis. Psychiatry Res..

[14] Tan N., van Os J. (2013). The schizophrenia spectrum and other psychotic disorders in the DSM-5. Tijdschr. Psychiatr..

[15] First M.B. (1994). Diagnostic and Statistical Manual of Mental Disorders.

[16] Brewerton T.D. (1994). Hyperreligiosity in psychotic disorders. J. Nerv. Ment. Dis..

[17] Kay S.R., Flszbein A., Opfer L.A. (1987). The positive and negative syndrome scale (PANSS) for schizophrenia. Schizophr. Bull..

[18] Hoch P.H. (1951). Experimentally produced psychoses. Am. J. Psychiatry.

[19] Gouzoulis-Mayfrank E., Habermeyer E., Hermle L., Steinmeyer A., Kunert H., Sass H. (1998). Hallucinogenic drug induced states resemble acute endogenous psychoses: Results of an empirical study. Eur. Psychiatry.

[20] Weil-Malherbe H., Szara S.I. (1971). The biochemistry of functional and experimental psychoses. J. Pharm. Sci..

[21] Unger S. (1964). The Current Scientific Status of Psychedelic Drug Research.

[22] Klee G., Weintraub W. (1959). Paranoid response following lysergic acid diethylamide (LSD-25). Neuro-Psychopharmacology.

[23] Langs R.J., Barr H.L. (1968). lysergic acid diethylamide (LSD-25) and schizophrenic reactions. J. Nerv. Ment. Dis..

[24] Anastasopoulos G., Photiades H. (1962). Effects of LSD-25 on relatives of schizophrenic patients. Br. J. Psychiatry.

[25] Power R.A., Verweij K.J., Zuhair M., Montgomery G.W., Henders A.K., Heath A.C., Madden P.A., Medland S.E., Wray N.R., Martin N.G. (2014). Genetic predisposition to schizophrenia associated with increased use of cannabis. Mol. Psychiatry.

[26] Henquet C., di Forti M., Morrison P., Kuepper R., Murray R.M. (2008). Gene-environment interplay between cannabis and psychosis. Schizophr. Bull..

[27] Vardy M.M., Kay S.R. (1983). LSD Psychosis or LSD-Induced Schizophrenia?: A Multimethod Inquiry. Arch. Gen. Psychiatry.

[28] Ungerleider J.T., Fisher D.D., Fuller M., Caldwell A. (1968). The “bad trip”-the etiology of the adverse LSD reaction. Am. J. Psychiatry.

[29] Smart R.G., Jones D. (1970). Illicit LSD users: Their personality characteristics and psychopathology. J. Abnorm. Psychol..

[30] Tucker G.J., Hanover N., Quinlan D., Harrow M. (1972). Chronic hallucinogenic drug use and thought disturbance. Arch. Gen. Psychiatry.

[31] Stone M. (1973). Drug-related schizophrenic syndromes. Int. J. Psychiatry.

[32] Cohen S. (1966). A classification of LSD complications. Psychosomatics.

[33] Johansen P.-Ø., Krebs T.S. (2015). Psychedelics not linked to mental health problems or suicidal behavior: A population study. J. Psychopharmacol..

[34] Blacker K., Jones R.T., Stone G.C., Pfefferbaum D. (1968). Chronic users of LSD: The “acidheads”. Am. J. Psychiatry.

[35] McGlothlin W.H., Arnold D.O., Freedman D.X. (1969). Organicity measures following repeated LSD ingestion. Arch. Gen. Psychiatry.

[36] Abraham H.D. (1982). A chronic impairment of colour vision in users of LSD. Br. J. Psychiatry.

[37] Halpern J.H., Pope H.G. (2003). Hallucinogen persisting perception disorder: What do we know after 50 years?. Drug Alcohol Depend..

[38] Behan W., Bakheit A., Behan P., More I. (1991). The muscle findings in the neuroleptic malignant syndrome associated with lysergic acid diethylamide. J. Neurol. Neurosurg. Psychiatry.

[39] Carhart-Harris R., Kaelen M., Bolstridge M., Williams T., Williams L., Underwood R., Feilding A., Nutt D. (2016). The paradoxical psychological effects of lysergic acid diethylamide (LSD). Psychol. Med..

[40] Giannini A.J., Eighan M.S., Loiselle R.H., Giannini M.C. (1984). Comparison of haloperidol and chlorpromazine in the treatment of phencyclidine psychosis. J. Clin. Pharmacol..

[41] Marona-Lewicka D., Nichols C.D., Nichols D.E. (2011). An animal model of schizophrenia based on chronic LSD administration: Old idea, new results. Neuropharmacology.

[42] Carhart-Harris R.L., Muthukumaraswamy S., Roseman L., Kaelen M., Droog W., Murphy K., Tagliazucchi E., Schenberg E.E., Nest T., Orban C. (2016). Neural correlates of the LSD experience revealed by multimodal neuroimaging. Proc. Natl. Acad. Sci. USA.

[43] Schneider K. (1959). Clinical Psychopathology.

[44] Steeds H., Carhart-Harris R.L., Stone J.M. (2015). Drug models of schizophrenia. Ther. Adv. Psychopharmacol..

[45] Woolley D., Shaw E. (1954). Some neurophysiological aspects of serotonin. Br. Med. J..

[46] Shaw E., Woolley D. (1956). Some serotoninlike activities of lysergic acid diethylamide. Science.

[47] Nichols C.D., Sanders-Bush E. (2004). Molecular genetic responses to lysergic acid diethylamide include transcriptional activation of MAP kinase phosphatase-1, C/EBP-β and ILAD-1, a novel gene with homology to arrestins. J. Neurochem..

[48] Halberstadt A.L., Geyer M.A. (2013). Serotonergic hallucinogens as translational models relevant to schizophrenia. Int. J. Neuropsychopharmacol..

[49] Sanders-Bush E., Burris K.D., Knoth K. (1988). Lysergic acid diethylamide and 2, 5-dimethoxy-4-methylamphetamine are partial agonists at serotonin receptors linked to phosphoinositide hydrolysis. J. Pharmacol. Exp. Ther..

[50] Rickli A., Luethi D., Reinisch J., Buchy D., Hoener M.C., Liechti M.E. (2015). Receptor interaction profiles of novel N-2-methoxybenzyl (NBOMe) derivatives of 2, 5-dimethoxy-substituted phenethylamines (2C drugs). Neuropharmacology.

[51] Rickli A., Moning O.D., Hoener M.C., Liechti M.E. (2016). Receptor interaction profiles of novel psychoactive tryptamines compared with classic hallucinogens. Eur. Neuropsychopharmacol..

[52] Norman A., Battaglia G., Creese I. (1985). [3H] WB4101 labels the 5-HT1A serotonin receptor subtype in rat brain. Guanine nucleotide and divalent cation sensitivity. Mol. Pharmacol..

[53] Reissig C., Eckler J., Rabin R., Winter J. (2005). The 5-HT1A receptor and the stimulus effects of LSD in the rat. Psychopharmacology.

[54] BURT D.R., Creese I., Snyder S.H. (1976). Binding interactions of lysergic acid diethylamide and related agents with dopamine receptors in the brain. Mol. Pharmacol..

[55] Aghajanian G.K., Marek G.J. (2000). Serotonin model of schizophrenia: Emerging role of glutamate mechanisms. Brain Res. Rev..

[56] Seeman P., Ko F., Tallerico T. (2005). Dopamine receptor contribution to the action of PCP, LSD and ketamine psychotomimetics. Mol. Psychiatry.

[57] Minuzzi L., Nomikos G.G., Wade M.R., Jensen S.B., Olsen A.K., Cumming P. (2005). Interaction between LSD and dopamine D2/3 binding sites in pig brain. Synapse.

[58] Seeman P., Guan H.C., Hirbec H. (2009). Dopamine D2High receptors stimulated by phencyclidines, lysergic acid diethylamide, salvinorin A, and modafinil. Synapse.

[59] Bunzow J.R., Sonders M.S., Arttamangkul S., Harrison L.M., Zhang G., Quigley D.I., Darland T., Suchland K.L., Pasumamula S., Kennedy J.L. (2001). Amphetamine, 3,4-methylenedioxymethamphetamine, lysergic acid diethylamide, and metabolites of the catecholamine neurotransmitters are agonists of a rat trace amine receptor. Mol. Pharmacol..

[60] Lindemann L., Meyer C.A., Jeanneau K., Bradaia A., Ozmen L., Bluethmann H., Bettler B., Wettstein J.G., Borroni E., Moreau J.-L. (2008). Trace amine-associated receptor 1 modulates dopaminergic activity. J. Pharmacol. Exp. Ther..

[61] Xie Z., Miller G.M. (2007). Trace amine-associated receptor 1 is a modulator of the dopamine transporter. J. Pharmacol. Exp. Ther..

[62] Revel F., Moreau J., Pouzet B., Mory R., Bradaia A., Buchy D., Metzler V., Chaboz S., Zbinden K.G., Galley G. (2013). A new perspective for schizophrenia: TAAR1 agonists reveal antipsychotic-and antidepressant-like activity, improve cognition and control body weight. Mol. Psychiatry.

[63] Davis K.L., Kahn R.S., Ko G., Davidson M. (1991). Dopamine in schizophrenia: A review and reconceptualization. Am. J. Psychiatry.

[64] Seeman P., Weinshenker D., Quirion R., Srivastava L.K., Bhardwaj S.K., Grandy D.K., Premont R.T., Sotnikova T.D., Boksa P., El-Ghundi M. (2005). Dopamine supersensitivity correlates with D2^High^ states, implying many paths to psychosis. Proc. Natl. Acad. Sci. USA.

[65] Sax K.W., Strakowski S.M., Keck P., Upadhyaya V.H., West S.A., McELROY S.L. (1996). Relationships among negative, positive, and depressive symptoms in schizophrenia and psychotic depression. Br. J. Psychiatry.

[66] Butler R.W., Mueser K.T., Sprock J., Braff D.L. (1996). Positive symptoms of psychosis in posttraumatic stress disorder. Biol. Psychiatry.

[67] Zimmermann G., Favrod J., Trieu V., Pomini V. (2005). The effect of cognitive behavioral treatment on the positive symptoms of schizophrenia spectrum disorders: A meta-analysis. Schizophr. Res..

[68] Kringelbach M.L., Berridge K.C. (2009). Towards a functional neuroanatomy of pleasure and happiness. Trends Cogn. Sci..

[69] Farde L., Nordström A.-L., Wiesel F.-A., Pauli S., Halldin C., Sedvall G. (1992). Positron emission tomographic analysis of central D1 and D2 dopamine receptor occupancy in patients treated with classical neuroleptics and clozapine: Relation to extrapyramidal side effects. Arch. Gen. Psychiatry.

[70] Nordstrom A.-L., Farde L. (1998). Plasma prolactin and central D2 receptor occupancy in antipsychotic drug-treated patients. J. Clin. Psychopharmacol..

[71] Kapur S., Remington G. (1996). Serotonin-dopamine interaction and its relevance to schizophrenia. Am. J. Psychiatry.

[72] Meltzer H., Massey B. (2011). The role of serotonin receptors in the action of atypical antipsychotic drugs. Curr. Opin. Pharmacol..

[73] Daskalakis Z., Christensen B., Zipursky R., Zhang-Wong J., Beiser M. (1998). 376. Relationship between D2 occupancy and prolactin levels in first episode psychosis. Biol. Psychiatry.

[74] Davis J.M., Chen N., Glick I.D. (2003). A meta-analysis of the efficacy of second-generation antipsychotics. Arch. Gen. Psychiatry.

[75] Leucht S., Wahlbeck K., Hamann J., Kissling W. (2003). New generation antipsychotics versus low-potency conventional antipsychotics: A systematic review and meta-analysis. Lancet.

[76] Artigas F. (2010). The prefrontal cortex: A target for antipsychotic drugs. Acta Psychiatr. Scand..

[77] Vázquez-Borsetti P., Cortés R., Artigas F. (2009). Pyramidal neurons in rat prefrontal cortex projecting to ventral tegmental area and dorsal raphe nucleus express 5-HT2A receptors. Cereb. Cortex.

[78] Díaz-Mataix L., Scorza M.C., Bortolozzi A., Toth M., Celada P., Artigas F. (2005). Involvement of 5-HT1A receptors in prefrontal cortex in the modulation of dopaminergic activity: Role in atypical antipsychotic action. J. Neurosci..

[79] Rollema H., Lu Y., Schmidt A.W., Zorn S.H. (1997). Clozapine increases dopamine release in prefrontal cortex by 5-HT 1A receptor activation. Eur. J. Pharmacol..

[80] Millan M.J. (2000). Improving the treatment of schizophrenia: Focus on serotonin (5-HT) 1A receptors. J. Pharmacol. Exp. Ther..

[81] Bantick R., Deakin J., Grasby P. (2001). The 5-HT1A receptor in schizophrenia: A promising target for novel atypical neuroleptics?. J. Psychopharmacol..

[82] Sumiyoshi T., Park S., Jayathilake K., Roy A., Ertugrul A., Meltzer H.Y. (2007). Effect of buspirone, a serotonin 1A partial agonist, on cognitive function in schizophrenia: A randomized, double-blind, placebo-controlled study. Schizophr. Res..

[83] Wadenberg M.-L. (1992). Antagonism by 8-OH-DPAT, but not ritanserin, of catalepsy induced by SCH 23390 in the rat. J. Neural Transm. Gen. Sect..

[84] Hicks P.B. (1990). The effect of serotonergic agents on haloperidol-induced catalepsy. Life Sci..

[85] Maj J., Sarnek J., Klimek V., Rawlow A. (1976). On the anticataleptic action of cyproheptadine. Pharmacol. Biochem. Behav..

[86] Fuenmayor L.D., Vogt M. (1979). the influence of cerebral 5-hydroxytryptamine on catalepsy induced by brain-amine depleting neuroleptics or by cholinomimetics. Br. J. Pharmacol..

[87] Yamaguchi K., Nabeshima T., Kameyama T. (1986). Potentiation of phencyclidine-induced dopamine-dependent behaviors in rats after pretreatments with serotonin-depletors. J. Pharmacobiodyn..

[88] Baldessarini R.J., Amatruda T.T., Griffith F.F., Gerson S. (1975). Differential effects of serotonin on turning and stereotypy induced by apomorphine. Brain Res..

[89] Kapur S., Seeman P. (2001). Does fast dissociation from the dopamine D2 receptor explain the action of atypical antipsychotics?: A new hypothesis. Am. J. Psychiatry.

[90] Cox S.M., Benkelfat C., Dagher A., Delaney J.S., Durand F., McKenzie S.A., Kolivakis T., Casey K.F., Leyton M. (2009). Striatal dopamine responses to intranasal cocaine self-administration in humans. Biol. Psychiatry.

[91] Stockton M.E., Rasmussen K. (1996). Electrophysiological effects of olanzapine, a novel atypical antipsychotic, on A9 and A10 dopamine neurons. Neuropsychopharmacology.

[92] van Domburg P.H.M.F., ten Donkelaar H.J. (1991). The Human Substantia Nigra and Ventral Tegmental Area. The Human Substantia Nigra and Ventral Tegmental Area.

[93] Borowsky B., Adham N., Jones K.A., Raddatz R., Artymyshyn R., Ogozalek K.L., Durkin M.M., Lakhlani P.P., Bonini J.A., Pathirana S. (2001). Trace amines: Identification of a family of mammalian G protein-coupled receptors. Proc. Natl. Acad. Sci. USA.

[94] Revel F.G., Moreau J.-L., Gainetdinov R.R., Bradaia A., Sotnikova T.D., Mory R., Durkin S., Zbinden K.G., Norcross R., Meyer C.A. (2011). TAAR1 activation modulates monoaminergic neurotransmission, preventing hyperdopaminergic and hypoglutamatergic activity. Proc. Natl. Acad. Sci. USA.

[95] Grandy D.K. (2007). Trace amine-associated receptor 1—Family archetype or iconoclast?. Pharmacol. Ther..

[96] Lindemann L., Hoener M.C. (2005). A renaissance in trace amines inspired by a novel GPCR family. Trends Pharmacol. Sci..

[97] Miller G.M. (2011). The emerging role of trace amine-associated receptor 1 in the functional regulation of monoamine transporters and dopaminergic activity. J. Neurochem..

[98] Narang D., Tomlinson S., Holt A., Mousseau D.D., Baker G.B. (2011). Trace amines and their relevance to psychiatry and neurology: A brief overview. Bull. Clin. Psychopharmacol..

[99] Sotnikova T.D., Caron M.G., Gainetdinov R.R. (2009). Trace amine-associated receptors as emerging therapeutic targets. Mol. Pharmacol..

[100] Berry M. (2007). The potential of trace amines and their receptors for treating neurological and psychiatric diseases. Rev. Recent Clin. Trials.

[101] Burchett S.A., Hicks T.P. (2006). The mysterious trace amines: Protean neuromodulators of synaptic transmission in mammalian brain. Prog. Neurobiol..

[102] Wolinsky T., Swanson C., Smith K., Zhong H., Borowsky B., Seeman P., Branchek T., Gerald C. (2007). The Trace Amine 1 receptor knockout mouse: An animal model with relevance to schizophrenia. Genes Brain Behav..

[103] Bradaia A., Trube G., Stalder H., Norcross R.D., Ozmen L., Wettstein J.G., Pinard A., Buchy D., Gassmann M., Hoener M.C. (2009). The selective antagonist EPPTB reveals TAAR1-mediated regulatory mechanisms in dopaminergic neurons of the mesolimbic system. Proc. Natl. Acad. Sci. USA.

[104] Stalder H., Hoener M.C., Norcross R.D. (2011). Selective antagonists of mouse trace amine-associated receptor 1 (mTAAR1): Discovery of EPPTB (RO5212773). Bioorg. Med. Chem. Lett..

[105] Espinoza S., Salahpour A., Masri B., Sotnikova T.D., Messa M., Barak L.S., Caron M.G., Gainetdinov R.R. (2011). Functional interaction between trace amine-associated receptor 1 and dopamine D2 receptor. Mol. Pharmacol..

[106] Glennon R.A., Titeler M., McKenney J. (1984). Evidence for 5-HT2 involvement in the mechanism of action of hallucinogenic agents. Life Sci..

[107] Teitler M., Leonhardt S., Appel N.M., Souza E.B., Glennon R.A. (1990). Receptor pharmacology of MDMA and related hallucinogensa. Ann. N. Y. Acad. Sci..

[108] Nichols D.E., Frescas S., Marona-Lewicka D., Huang X., Roth B.L., Gudelsky G.A., Nash J.F. (1994). 1-(2, 5-Dimethoxy-4-(trifluoromethyl) phenyl)-2-aminopropane: A potent serotonin 5-HT2A/2C agonist. J. Med. Chem..

[109] Kometer M., Schmidt A., Jäncke L., Vollenweider F.X. (2013). Activation of serotonin 2A receptors underlies the psilocybin-induced effects on α oscillations, N170 visual-evoked potentials, and visual hallucinations. J. Neurosci..

[110] Rasmussen K., Aghajanian G.K. (1986). Effect of hallucinogens on spontaneous and sensory-evoked locus coeruleus unit activity in the rat: Reversal by selective 5-HT2antagonists. Brain Res..

[111] Geyer M.A., Krebs K.M. (1994). Serotonin receptor involvement in an animal model of the acute effects of hallucinogens. NIDA Res. Monogr..

[112] Marek G.J., Aghajanian G.K. (1996). LSD and the phenethylamine hallucinogen DOI are potent partial agonists at 5-HT2A receptors on interneurons in rat piriform cortex. J. Pharmacol. Exp. Ther..

[113] Sanders-Bush E., Breeding M. (1991). Choroid plexus epithelial cells in primary culture: A model of 5HT1C receptor activation by hallucinoginic drugs. Psychopharmacology.

[114] Egan C.T., Herrick-Davis K., Miller K., Glennon R.A., Teitler M. (1998). Agonist activity of LSD and lisuride at cloned 5HT2A and 5HT2C receptors. Psychopharmacology.

[115] De Gregorio D., Posa L., Ochoa-Sanchez R., McLaughlin R., Maione S., Comai S., Gobbi G. (2016). The hallucinogen d-lysergic diethylamide (LSD) decreases dopamine firing activity through 5-HT1A, D2 and TAAR1 receptors. Pharmacol. Res..

[116] Fiorella D., Helsley S., Lorrain D., Rabin R.A., Winter J. (1995). The role of the 5-HT2A and 5-HT2C receptors in the stimulus effects of hallucinogenic drugs III: The mechanistic basis for supersensitivity to the LSD stimulus following serotonin depletion. Psychopharmacology.

[117] Paxinos G., Watson C. (1986). The Rat Brain in Stereotaxic Coordinates.

[118] Krall C., Richards J., Rabin R., Winter J. (2008). Marked decrease of LSD-induced stimulus control in serotonin transporter knockout mice. Pharmacol. Biochem. Behav..

[119] Kyzar E.J., Stewart A.M., Kalueff A.V. (2016). Effects of LSD on grooming behavior in serotonin transporter heterozygous (*Sert*^+/−^) mice. Behav. Brain Res..

[120] Watts V.J., Mailman R., Lawler C., Neve K.A., Nichols D.E. (1995). LSD and structural analogs: Pharmacological evaluation at D1 dopamine receptors. Psychopharmacology.

[121] Appel J., White F., Holohean A. (1983). Analyzing mechanism(s) of hallucinogenic drug action with drug discrimination procedures. Neurosci. Biobehav. Rev..

[122] Meert T., de Haes P., Janssen P. (1989). Risperidone (R 64 766), a potent and complete LSD antagonist in drug discrimination by rats. Psychopharmacology.

[123] Marona-Lewicka D., Thisted R.A., Nichols D.E. (2005). Distinct temporal phases in the behavioral pharmacology of LSD: Dopamine D2 receptor-mediated effects in the rat and implications for psychosis. Psychopharmacology.

[124] Marona-Lewicka D., Chemel B.R., Nichols D.E. (2009). Dopamine D4 receptor involvement in the discriminative stimulus effects in rats of LSD, but not the phenethylamine hallucinogen DOI. Psychopharmacology.

[125] Lambe E.K., Aghajanian G.K. (2006). Hallucinogen-induced UP states in the brain slice of rat prefrontal cortex: Role of glutamate spillover and NR2B-NMDA receptors. Neuropsychopharmacology.

[126] Moreno J.L., Holloway T., Rayannavar V., Sealfon S.C., González-Maeso J. (2013). Chronic treatment with LY341495 decreases 5-HT 2A receptor binding and hallucinogenic effects of LSD in mice. Neurosci. Lett..

[127] Giacomelli S., Palmery M., Romanelli L., Cheng C.Y., Silvestrini B. (1998). Lysergic acid diethylamide (LSD) is a partial agonist of D2 dopaminergic receptors and it potentiates dopamine-mediated prolactin secretion in lactotrophs in vitro. Life Sci..

[128] Meltzer H.Y., Fessler R.G., Simonovic M., Doherty J., Fang V.S. (1977). Lysergic acid diethylamide: Evidence for stimulation of pituitary dopamine receptors. Psychopharmacology.

[129] Meert T., Awouters F. (1990). Central 5-HT2 antagonists: A preclinical evaluation of a therapeutic potential. Acta Neuropsychiatr..

[130] Koek W., Jackson A., Colpaert F.C. (1992). Behavioral pharmacology of antagonists at 5-HT2/5-HT1C receptors. Neurosci. Biobehav. Rev..

[131] Meert T. (1996). Serotonin Mechanisms in Antipsychotic Treatment: Evidence from Drug Discrimination Studies. Serotonin in Antipsychotic Treatment. Mechanisms and Clinical Practice.

[132] Marona-Lewicka D., Nichols D.E. (2007). Further evidence that the delayed temporal dopaminergic effects of LSD are mediated by a mechanism different than the first temporal phase of action. Pharmacol. Biochem. Behav..

[133] Vollenweider F.X., Vollenweider-Scherpenhuyzen M.F., Bäbler A., Vogel H., Hell D. (1998). Psilocybin induces schizophrenia-like psychosis in humans via a serotonin-2 agonist action. Neuroreport.

[134] Martin D.A., Marona-Lewicka D., Nichols D.E., Nichols C.D. (2014). Chronic LSD alters gene expression profiles in the mPFC relevant to schizophrenia. Neuropharmacology.

[135] Pehek E.A., Nocjar C., Roth B.L., Byrd T.A., Mabrouk O.S. (2006). Evidence for the preferential involvement of 5-HT2A serotonin receptors in stress-and drug-induced dopamine release in the rat medial prefrontal cortex. Neuropsychopharmacology.

[136] Vollenweider F., Leenders K., Scharfetter C., Maguire P., Stadelmann O., Angst J. (1997). Positron emission tomography and fluorodeoxyglucose studies of metabolic hyperfrontality and psychopathology in the psilocybin model of psychosis. Neuropsychopharmacology.

[137] Simmler L.D., Buchy D., Chaboz S., Hoener M.C., Liechti M.E. (2016). In vitro characterization of psychoactive substances at rat, mouse, and human trace amine-associated receptor 1. J. Pharmacol. Exp. Ther..

[138] Chandler D.J., Lamperski C.S., Waterhouse B.D. (2013). Identification and distribution of projections from monoaminergic and cholinergic nuclei to functionally differentiated subregions of prefrontal cortex. Brain Res..

[139] Steketee J.D. (2003). Neurotransmitter systems of the medial prefrontal cortex: Potential role in sensitization to psychostimulants. Brain Res. Rev..

[140] Arvanov V.L., Liang X., Russo A., Wang R.Y. (1999). LSD and DOB: Interaction with 5-HT2A receptors to inhibit NMDA receptor-mediated transmission in the rat prefrontal cortex. Eur. J. Neurosci..

[141] Solinas M., Panlilio L.V., Justinova Z., Yasar S., Goldberg S.R. (2006). Using drug-discrimination techniques to study the abuse-related effects of psychoactive drugs in rats. Nat. Protoc..

[142] Geyer M.A., Swerdlow N.R., Mansbach R.S., Braff D.L. (1990). Startle response models of sensorimotor gating and habituation deficits in schizophrenia. Brain Res. Bull..

[143] Geyer M.A. (1998). Behavioral studies of hallucinogenic drugs in animals: Implications for schizophrenia research. Pharmacopsychiatry.

[144] Meltzer H.Y., Matsubara S., Lee J. (1989). Classification of typical and atypical antipsychotic drugs on the basis of dopamine D-1, D-2 and serotonin2 pKi values. J. Pharmacol. Exp. Ther..

[145] Geyer M.A., Krebs-Thomson K., Braff D.L., Swerdlow N.R. (2001). Pharmacological studies of prepulse inhibition models of sensorimotor gating deficits in schizophrenia: A decade in review. Psychopharmacology.

[146] Braff D.L., Swerdlow N.R., Geyer M.A. (1999). Symptom correlates of prepulse inhibition deficits in male schizophrenic patients. Am. J. Psychiatry.

[147] Parwani A., Duncan E.J., Bartlett E., Madonick S.H., Efferen T.R., Rajan R., Sanfilipo M., Chappell P.B., Chakravorty S., Gonzenbach S. (2000). Impaired prepulse inhibition of acoustic startle in schizophrenia. Biol. Psychiatry.

[148] Quednow B.B., Frommann I., Berning J., Kühn K.-U., Maier W., Wagner M. (2008). Impaired sensorimotor gating of the acoustic startle response in the prodrome of schizophrenia. Biol. Psychiatry.

[149] Ouagazzal A., Grottick A., Moreau J., Higgins G. (2001). Effect of LSD on prepulse inhibition and spontaneous behavior in the rat: A pharmacological analysis and comparison between two rat strains. Neuropsychopharmacology.

[150] Halberstadt A.L., Geyer M.A. (2010). LSD but not lisuride disrupts prepulse inhibition in rats by activating the 5-HT2A receptor. Psychopharmacology.

[151] Canal C.E., Morgan D. (2012). Head-twitch response in rodents induced by the hallucinogen 2, 5-dimethoxy-4-iodoamphetamine: A comprehensive history, a re-evaluation of mechanisms, and its utility as a model. Drug Test. Anal..

[152] González-Maeso J., Yuen T., Ebersole B.J., Wurmbach E., Lira A., Zhou M., Weisstaub N., Hen R., Gingrich J.A., Sealfon S.C. (2003). Transcriptome fingerprints distinguish hallucinogenic and nonhallucinogenic 5-hydroxytryptamine 2A receptor agonist effects in mouse somatosensory cortex. J. Neurosci..

[153] Benneyworth M.A., Smith R.L., Sanders-Bush E. (2008). Chronic phenethylamine hallucinogen treatment alters behavioral sensitivity to a metabotropic glutamate 2/3 receptor agonist. Neuropsychopharmacology.

[154] Halberstadt A.L., Geyer M.A. (2013). Characterization of the head-twitch response induced by hallucinogens in mice. Psychopharmacology.

[155] Fantegrossi W.E., Harrington A.W., Eckler J.R., Arshad S., Rabin R.A., Winter J.C., Coop A., Rice K.C., Woods J.H. (2005). Hallucinogen-like actions of 2, 5-dimethoxy-4-(*n*)-propylthiophenethylamine (2C-T-7) in mice and rats. Psychopharmacology.

[156] Fribourg M., Moreno J.L., Holloway T., Provasi D., Baki L., Mahajan R., Park G., Adney S.K., Hatcher C., Eltit J.M. (2011). Decoding the signaling of a GPCR heteromeric complex reveals a unifying mechanism of action of antipsychotic drugs. Cell.

[157] Moreno J.L., Holloway T., Umali A., Rayannavar V., Sealfon S.C., González-Maeso J. (2013). Persistent effects of chronic clozapine on the cellular and behavioral responses to LSD in mice. Psychopharmacology.

[158] Tófoli L., de Araujo D. (2016). Chapter Seven-Treating Addiction: Perspectives from EEG and Imaging Studies on Psychedelics. Int. Rev. Neurobiol..

[159] Dos Santos R.G., Osório F.L., Crippa J.A.S., Riba J., Zuardi A.W., Hallak J.E. (2016). Antidepressive, anxiolytic, and antiaddictive effects of ayahuasca, psilocybin and lysergic acid diethylamide (LSD): A systematic review of clinical trials published in the last 25 years. Ther. Adv. Psychopharmacol..

[160] Gasser P., Holstein D., Michel Y., Doblin R., Yazar-Klosinski B., Passie T., Brenneisen R. (2014). Safety and efficacy of lysergic acid diethylamide-assisted psychotherapy for anxiety associated with life-threatening diseases. J. Nerv. Ment. Dis..

[161] Dolder P.C., Schmid Y., Müller F., Borgwardt S., Liechti M.E. (2016). LSD acutely impairs fear recognition and enhances emotional empathy and sociality. Neuropsychopharmacology.

